# *In vitro* protoscolicidal effects of *Hypericum perforatum, Thymus vulgaris, Pimenta racemosa*, and *Mentha piperita* against *Echinococcus granulosus* protoscoleces

**DOI:** 10.2478/helm-2025-0021

**Published:** 2025-09-30

**Authors:** R. Yildiz, A. H. Unlu

**Affiliations:** Division of Veterinary, Vocational School of Gevas, Van Yuzuncu Yil University, Van, Turkiye

**Keywords:** Echinococcus granulosus, Hypericum perforatum, Mentha piperita, Pimenta racemose, Protoscolicidal, Thymus vulgaris

## Abstract

Cystic echinococcosis (CE), an endemic zoonotic disease in Turkiye, is caused by the helminth *Echinococcus granulosus*. It has threatened the livestock industry and is of major medical and socio-economic importance to humans. Surgery is still the main practice of treatment, despite the risk of relapses and several adverse effects. Due to their minimal side effects, herbal products have been widely used for thousands of years to treat various infections. The present study was designed to investigate the *in vitro* protoscolicidal effect of extracts from *Hypericum perforatum, Thymus vulgaris, Pimenta racemosa*, and *Mentha piperita* against hydatid cyst protoscoleces. The livers and lungs of the sheep were obtained from a private abattoir, and the protoscoleces were collected under sterile conditions. The fi nal herbal products were extracted using the aqueous extraction method. Different concentrations of herbal extracts (50, 100, and 150 mg/ml) were tested on *Echinococcus granulosus* protoscoleces, following different times of incubation (5, 10, and 60 min). The viability of the protoscoleces was assessed by a 0.1 % eosin staining method. Extracts of *H. perforatum, T. vulgaris*, and *P. racemosa* exhibited a statistically signifi cant effect on protoscoleces mortality (P<0.001) when compared with the negative control. The present experimental study indicated that aqueous extracts of *H. perforatum, T. vulgaris*, and *P. racemosa* could be potential candidates as valuable and useful sources of protoscolicidal agents for CE.

## Introduction

Cystic echinococcosis (CE), a zoonotic parasitic disease, is caused by the metacestode of the canine larval tapeworm *Echinococcus granulosus*. The infection results from the development of hydatid cysts, especially in the liver and lungs, although cysts can form in any part of the body (Budke *et al*., 2009). CE infestation results in economic losses, including carcass condemnation, reduced milk production and fertility, as well as increased weight loss and mortality (Bekele & Butako, 2011; Jairus *et al*., 2023). Economic losses include medical and veterinary costs, which have been estimated at around $3 billion a year (WHO, 2024).

Currently, parasitic diseases are controlled by synthetic drugs, which have signifi cant negative impacts such as their high cost, the potential for drug resistance and their environmental impact. As a result, new therapies, such as those using traditional medicinal plants, are becoming increasingly popular (Kheirandish *et al*., 2020). However, these medicinal plants require further study regarding their characteristics, safety, and effects (Nascimento *et al*., 2000). In this context, researchers are currently seeking new, safer, and more effective protoscolicidal agents for the treatment of CE (Alvi *et al*., 2022).

Herbal medicinal products such as *Olea europaea* (Zibaei *et al*., 2012), *Berberis vulgaris* (Rouhani *et al*., 2013), *Zingiber officinale* (Amri & Touil-Boukoffa, 2016*), Blepharocalyx salicifolius* (Noal *et al*., 2017), *Artemisia sieberi* (Vakili *et al*., 2019), *Punica granatum* (Labsi *et al*., 2019), *Atriplex halimus* (Bouaziz *et al*., 2021), *Malva sylvestris* (Jasim, 2023), *Juniperus phoenicea* (Al-khlifeh *et al*., 2023), *Calotropis procera* (Al-khlifeh *et al*., 2023), and *Artemisia judaica* (Al-khlifeh *et al*., 2023) have shown promising effects on killing protoscoleces *in vivo* and/or *in vitro*.

*Hypericum perforatum* is a plant widely used in traditional medicine, and its antimicrobial properties have been demonstrated. (Kladar *et al*., 2017; Sherif *et al*., 2023). *Thymus vulgaris* possesses several medicinal properties, including anticoccidial (Jamroz *et al*., 2003) and anthelmintic (Rasooli *et al*., 2006) effects. It contains many substances with therapeutic value, such as thymol, eugenol, flavonoids, and others (Amarowicz *et al*., 2008). *Pimenta racemose* has traditionally been used for medicinal purposes, and its antibacterial effect has previously been shown (Deberdt *et al*., 2018). A study conducted on the antimicrobial activity of *Mentha piperita* essential oils showed that it has the potential to control several pathogens such as fungi and bacteria (Camele *et al*., 2021).

The aqueous extraction method is a valuable traditional practice. It provides a safe and effective way of extracting medicinal compounds from plants. This technique can be very efficient for the extraction of water-soluble phytochemicals, such as flavonoids, tannins, and alkaloids (Sarikurkcu *et al*., 2020).

In this present study, we evaluated the *in vitro* protoscolicidal effect of *Hypericum perforatum, Thymus vulgaris, Pimenta racemosa*, and *Mentha piperita* against *Echinococcus granulosus* protoscoleces using the aqueous extraction method.

## Material and Methods

### Collection of protoscoleces and screening their viability

Cysts were collected from the livers and lungs of locally infected sheep that had been slaughtered in a private operator’s slaughterhouse in Van, Turkey. The hydatid fluid was poured into a small beaker and left to settle for 30 min to allow the protoscoleces to precipitate. The upper layer was gently removed, and the precipitated protoscoleces were rinsed three times in 0.9 % sodium chloride. The viability of the protoscoleces was confirmed by a 0.1 % eosin exclusion test under the light microscope (Leica DM500).

### Aqueous extracts of the plant samples

Plants were obtained from the seller of medicinal herbs in the center of Van province, Turkiye. Plant parts were washed carefully and then air-dried. Once air-dried, the samples were pulverized in a mortar, and 40 g of each crumbled sample was poured into a glass beaker containing 160 mL of dH_2_O. Samples were incubated at room temperature for 72 h. Then, the filtration process was performed using glass filter funnels and filter papers. The filtered samples were evaporated for the condensation process and dried in sterilizator (Termal Laboratory, Type: G11420SD, Holland) at 45 °C. The samples were then stored at 4°C for later processing.

### In vitro assessment of the final product

Three concentrations, 50, 100, and 150 mg/mL, were prepared by adding 1.5, 3, and 4.5 g of the final plant product, respectively, and dissolving them in 30 mL PBS buffer in tubes. Protoscoleces were mixed with the prepared herbal extract at the desired concentration in a test tube and incubated for the specified time. The three concentrations of herbal extracts were tested for 5, 10, and 60 minutes against *E. granulosus* protoscoleces, and their protoscolicidal effect was evaluated using the eosin (0.1 %) exclusion test. A total of 150 *E. granulosus* protoscoleces were counted under the light microscope, and the number of viable and dead protoscoleces was immediately recorded. All tests were performed in triplicate, and the mean value was taken as the result. Hypertonic saline (20 %) was used as the positive control, and sodium chloride (0.9 %) was used as the negative control. Previous studies describing the active molecules in the aqueous extracts of the plant samples are mentioned in the discussion section, along with their corresponding references.

### Statistical analysis

Statistical analysis was carried out using SPSS software (version 21). Differences between the experimental and control groups were analyzed using one-way ANOVA with Tukey post-hoc tests. Data are represented as mean ± standard deviation (SD). Furthermore, P<0.001 was considered statistically significant.

## Ethical Approval and/or Informed Consent

This article does not contain any studies with human participants or animals.

## Results

**Fig. 1.**
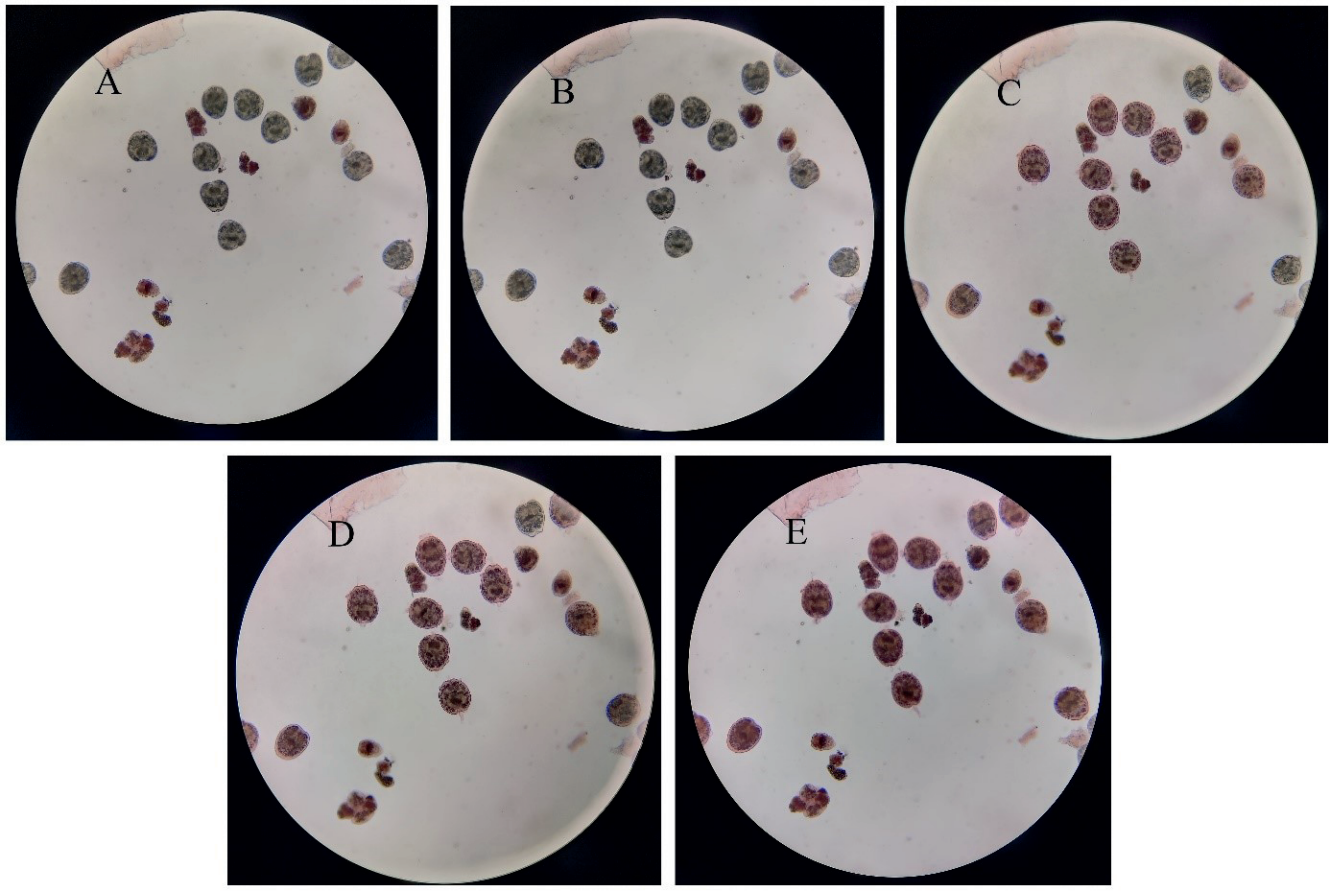
Effect of *T. vulgaris* extract (150 mg/ml) on protoscoleces. The same microscopic image was photographed at one-minute intervals to observe how the protoscoleces took up the 0.1% eosin after their death (A: min 1, B: min 2, C: min 3, D: min 4, E: min 5, and magnification: 40X).

*In vitro* protoscolicidal effects of 50, 100, and 150 mg/mL of herbal extracts were demonstrated after 5, 10, and 60 min of incubation (Table 1). Mortality rates on protoscoleces for 50, 100 and 150 mg/ml concentrations of *H. perforatum, T. vulgaris* and *P. racemosa* extracts following 10 min were 49.1 ± 1.35, 52.2 ± 2.7, 54.4 ± 1.35; 40.2 ± 2.19, 66.2 ± 3.64, 98 ± 1.15; and 52.2 ± 2.92, 76.9 ± 2.99, 96.8 ± 3.88, respectively. Mortality rates of *T. vulgaris* and *P. racemosa* at 100 mg/ml concentrations following 5 minutes were 60.9 ± 2.25 and 55.1 ± 3.95, respectively. Mortality rates of *T. vulgaris* and *P. racemosa* at 150 mg/ml concentrations following 5 minutes were 96.5 ± 2.58 and 60.2 ± 2.73, respectively. Mortality rates on protoscoleces for 50, 100 and 150 mg/ml concentrations of *Hypericum perforatum, Thymus vulgaris* and *Pimenta racemosa* extracts following 60 min were 88.9 ± 3.85, 91.6 ± 3.7, 93.3 ± 3.29; 56.9 ± 1.95, 99.3 ± 0.39, 99.8 ± 0.2; and 95.3 ± 2.31, 96.7 ± 1.76, 98.2 ± 0.97, respectively. The combined effect of higher concentration and longer exposure time leads to significantly higher mortality rates. These results showed that all three plant extracts had a statistically significant effect on protoscoleces mortality (P<0.001) when compared with the negative control. *Mentha piperita* extract showed significantly lower mortality rates compared to other herbal extracts, even at higher concentrations and longer exposure times. *In vitro* protoscolicidal activity of *M. piperita* extract was limited to approximately 20 % at the highest dose and exposure time. The *T. vulgaris* extract showed a more substantial effect on mortality rates compared to other herbal extracts, especially at higher concentrations and longer exposure times. The scolicidal effect of a 150 mg/mL *T. vulgaris* extract could be observed at one-minute intervals (Fig. 1). The positive and negative controls validated the reliability of the results.

**Table 1.** *In vitro* protoscolicidal activity of different concentrations of herbal extracts at 5, 10, and 60 min.

Experimental Groups	Samples	Concentration	Mortality rate (%) with following exp. time		
			5 min	10 min	60 min
Treatment with herbal extract	*H. perforatum* (Leaves and flowers)	50 mg/ml 100 mg/ml 150 mg/ml	36.4 ± 1.74 37.8 ± 1.98 38.9 ± 2.35	49.1 ± 1.35 52.2 ± 2.7 54.4 ± 1.35	88.9 ± 3.85 91.6 ± 3.7 93.3 ± 3.29
	*T. vulgaris* (Leaves)	50 mg/ml 100 mg/ml 150 mg/ml	33.7 ± 1.56 60.9 ± 2.25 96.5 ± 2.58	40.2 ± 2.19 66.2 ± 3.64 98 ± 1.15	56.9 ± 1.95 99.3± 0.39 99.8 ± 0.2
	*P. racemose* (Leaves)	50 mg/ml 100 mg/ml 150 mg/ml	44.6 ± 3.53 55.1 ± 3.95 60.2 ± 2.73	52.2 ± 2.92 76.9 ± 2.99 96.8 ± 3.88	95.3 ± 2.31 96.7 ± 1.76 98.2 ± 0.97
	*M. piperita* (Leaves)	50 mg/ml 100 mg/ml 150 mg/ml	10.44 ± 0.97 16 ± 1.77 17.3 ± 1.92	11.6 ± 1.98 16.9 ± 1.35 19.3 ± 2.03	14.2 ± 2.73 18.7 ± 1.15 20 ± 1.02
Positive Control	NaCl	20 %	100 ± 0	100 ± 0	100 ± 0
Negative Control	NaCl	0.90 %	2.2 ± 0.46	3.3 ± 0.53	4.5 ± 0.97

## Discussion

Herbal medicines have long played a central role in complementary and alternative medicine worldwide (Kohansal *et al*., 2017). Medicinal plants, due to their minimal side effects, low cost, and wide availability, have become a source of new drugs for treating various diseases. There has been an increasing amount of research into herbal therapies and their beneficial effects against parasitic diseases. Nevertheless, it should not be ignored that the global trend in research is about to change, as the focus shifts from growing plants with medicinal properties to finding new drugs (Salmerón-Manzano *et al*., 2020).

Current treatments of CE include surgery, chemotherapy and percutaneous aspiration-injection-reaspiration. Moreover, albendazole and mebendazole are used as oral anthelmintics (Adas *et al*., 2010). However, there is often no complete recovery after treatment, and 40 % of patients do not achieve a sufficient response (Naguleswaran *et al*., 2006; Nicolao *et al*., 2014). Due to side effects, drug resistance and relapse of *Echinococcus granulosus* infestation following traditional therapies, the search for innovative therapeutic approaches, such as natural products, seems necessary (Boakye *et al*., 2023). It is therefore of vital importance to develop novel, safe, and efficient scolicidal agents.

A review of herbal medicines against hydatid disease revealed that methanolic extraction was the most commonly used extraction method in studies conducted between 2000 and 2021 (Alvi *et al*., 2022). This was followed by hydrodistillation and extraction methods using ethanolic and aqueous solvents, respectively. The ethanolic extract method had a stronger and faster scolicidal activity against *E. granulosus* protoscoleces compared to the aqueous extract. (Benmarce *et al*., 2024). However, aqueous extraction is a simple, environmentally friendly, safe and cost-effective method of extracting (El-Desouky, 2021).

It appears that there are very few *in vivo* studies on the effectiveness of medicinal plants against protoscoleces of *E. granulosus*. (Alvi *et al*., 2022). Protoscolicidal effects of *Punica granatum*’s peels were tested using an aqueous extraction method on mice (Labsi *et al*., 2016). A total of 16 mg/mL of plant extracts was applied for two days. Scolicidal efficacy was found to be 100 %. In a similar study conducted on mice, extracts from *Punica granatum* peels were used at a concentration of 0.65 mg/ml (Labsi *et al*., 2019). Plant extracts were applied for 60 days, and scolicidal efficacy was found to be 66.7 %. *Atriplex halimus* leaves were used against protoscoleces using the aqueous extract method, and mortality rates were 99.36 % and 100 % at 60 and 100 mg/ml, respectively, after 120 min exposure (Bouaziz et ark, 2021). The extracts had no cytotoxic effect on murine peritoneal macrophages. Several studies have investigated the *in vitro* efficacy of medicinal plants in treating *E. granulosus* protoscoleces using the aqueous extract method. *Olea europaea* leaf extracts were tested against the protoscoleces, resulting in a mortality rate of 96.7 % at 1 mg/mL after 120 minutes of incubation (Zibaei *et al*., 2012). *Berberis vulgaris* fruit extracts were used against the protoscoleces, and a 100 % mortality rate was observed at 4 mg/ml after 30 minutes of incubation (Rouhani *et al*., 2013). These results demonstrate that the scolicidal activity was highly effective at low concentrations and short exposure times. *Zingiber officinale* exhibited a time-dependent protoscolicidal effect at 24 h and 48 h, with maximum reductions of 89.72 % and 100 % (P < 0.0001) at 100 μg/ml, respectively (Amri & Touil-Boukoffa, 2016). *Blepharocalyx salicifolius* leaf extract (gallic acid) showed 100 % scolicidal activity against *Echinococcus ortleppi* at a concentration of 200 mg/ml for 5 min. (Noal *et al*., 2017). *Artemisia sieberi* extract showed 92.6 % ± 1.28 scolicidal activity against *E. granulosus* protoscoleces at a concentration of 75 mg/ml for 10 min (Vakili *et al*., 2019). The alcoholic and aqueous extracts of *Malva sylvestris* leaves had a 100 % protoscolocidal effect on *E. granulosus* protoscoleces at a concentration of 60 mg/ml for 60 min (Jasim, 2023). The anthelmintic effect of the essential oil of *Thymus vulgaris* against protoscoleces and cysts of *E. granulosus* has been previously shown (Pensel *et al*., 2014). In the present study, a total of four plants were examined for their protoscolocidal efficacy against the protoscoleces of *E. granulosus* (Table 1). The aqueous extracts of *Hypericum perforatum, Thymus vulgaris*, and *Pimenta racemose* had a significant effect on protoscolex mortality (P<0.001). The extract of *T. vulgaris* showed a more substantial effect on mortality rates compared to other groups, achieving near-complete mortality (up to 99.8 %) at higher concentrations and longer exposure times. Moreover, we thought it would be noteworthy to present photographically how rapidly the scolicidal effect of 150 mg/mL *T. vulgaris* extract occurs at one-minute intervals (Fig. 1).

A study conducted by Maggiore *et al*. (2012) showed a time-dependent anthelmintic activity of *Mentha* spp—essential oils on *E. granulosus* protoscoleces and metacestodes. The hydrodistillation extraction method was used in the study. *Mentha pulegium* had a much stronger anthelmintic efficacy than *M. piperita*. The scolicidal effect of *M. piperita* was slower, with nearly 50 % efficacy after 24 days. In our study, the *in vitro* protoscolicidal effect of *M. piperita* (150 mg/ml) was approximately 20 % in one hour. Considering the current and previous studies cited in the paper, it is clear that the scolicidal effect is dependent on both duration and dose in most cases.

*Hypericum perforatum* is characterized by the presence of several key bioactive compounds, including hyperoside (Chen *et al*., 2018), hesperidin (Hernández-Saavedra *et al*., 2016), quercetin (Chen *et al*., 2018), and chlorogenic acid (Mohammed *et al*., 2019), which are identified as its primary constituents. A study by Sarikurkcu *et al*. (2020) showed that the aqueous extract of *H. perforatum* contained a total of 26 phytochemical compounds, with hyperoside, hesperidin, quercetin, and chlorogenic acid as the major constituents, as determined by liquid chromatography with tandem mass spectrometry (LC-MS/MS) analysis. *Thymus vulgaris*, commonly known as thyme, is widely used for various medicinal applications. The essential oil of thyme is its active component, with thymol and carvacrol serving as the main compounds (Gavarić *et al*., 2015). The essential oil of *Pimenta racemosa* was extracted from the aerial parts by hydrodistillation and analysed by GC/MS. Forty-five compounds were identified, of which eugenol, β-pinene, linalool and limonene were the major components (Elshaarawy *et al*., 2024). Aqueous extracts of *M. piperita* leaves contain flavonoids such as eriocitrin, luteolin-7-O-glucoside and rosmarinic acid. While the highest amounts can be obtained with methanolic and acetonitrile extraction methods, low amounts can be obtained with the aqueous extraction method (Hudz *et al*., 2023). We believe that these bioactive compounds in each of the herbal extracts, either individually or in combination, are responsible for killing the parasite in our study. The current study only suggests the potential efficacy of the aqueous extracts. However, without *in vivo* (animal or clinical) studies, the actual therapeutic effectiveness remains uncertain.

In conclusion, our findings suggest that the aqueous extracts of *Hypericum perforatum, Thymus vulgaris*, and *Pimenta racemosa* could be a potential source of protoscolicidal agents, making them promising candidates for further studies. Additional *in vitro* and *in vivo* studies are needed to comprehensively evaluate the anthelmintic constituents of these plants for the therapy of cystic echinococcosis. Future studies may lead to exploring the mechanisms behind the herbal extract’s effectiveness and investigating compounds with enhanced therapeutic value.
